# Coregulation of host-adapted metabolism and virulence by pathogenic *yersiniae*

**DOI:** 10.3389/fcimb.2014.00146

**Published:** 2014-10-20

**Authors:** Ann Kathrin Heroven, Petra Dersch

**Affiliations:** Department of Molecular Infection Biology, Helmholtz Centre for Infection Research, Institut für Mikrobiology, Technische Universität BraunschweigBraunschweig, Germany

**Keywords:** *Yersinia*, host-adapted metabolism, virulence, gene regulation, Csr, Crp

## Abstract

Deciphering the principles how pathogenic bacteria adapt their metabolism to a specific host microenvironment is critical for understanding bacterial pathogenesis. The enteric pathogenic *Yersinia* species *Yersinia pseudotuberculosis* and *Yersinia enterocolitica* and the causative agent of plague, *Yersinia pestis*, are able to survive in a large variety of environmental reservoirs (e.g., soil, plants, insects) as well as warm-blooded animals (e.g., rodents, pigs, humans) with a particular preference for lymphatic tissues. In order to manage rapidly changing environmental conditions and interbacterial competition, *Yersinia* senses the nutritional composition during the course of an infection by special molecular devices, integrates this information and adapts its metabolism accordingly. In addition, nutrient availability has an impact on expression of virulence genes in response to C-sources, demonstrating a tight link between the pathogenicity of *yersiniae* and utilization of nutrients. Recent studies revealed that global regulatory factors such as the cAMP receptor protein (Crp) and the carbon storage regulator (Csr) system are part of a large network of transcriptional and posttranscriptional control strategies adjusting metabolic changes and virulence in response to temperature, ion and nutrient availability. Gained knowledge about the specific metabolic requirements and the correlation between metabolic and virulence gene expression that enable efficient host colonization led to the identification of new potential antimicrobial targets.

## Introduction

Intensive work on the virulence strategies of all three human pathogenic *Yersinia* species (*Yersinia pestis, Yersinia pseudotuberculosis*, and *Yersinia enterocolitica*) as well as studies on the molecular and cellular aspects of *Yersinia* pathogenesis have considerably increased our knowledge of how *yersiniae* establish infections and cause diseases. Over the last decades a large set of specific pathogenicity factors has been identified, which mediate efficient resistance against the host defense systems, manipulate host cell processes and enable the bacteria to colonize, invade and multiply in host tissues. The structure, function and regulation of many of those classical virulence factors have been characterized and their role in pathogenesis has been analyzed using different animal models. However, a simple additional premise for their fitness and success is that they are able to obtain nutrients (C-/N-/energy sources) and ions at the infection site. As a consequence, bacterial pathogens have evolved efficient host-adapted nutrient retrieval strategies to optimize their metabolism and maximize the harvest of essential ions, energy sources and biomass building blocks from tissues encountered during infection. Here, we will discuss recent advances in our knowledge of (i) nutritional adaptation strategies of human pathogenic *yersiniae* and (ii) molecular mechanisms dedicated to control and adjust metabolic processes with virulence functions.

## *Yersinia* life styles and pathogenesis

The genus *Yersinia* belongs to the family of *Enterobacteriaceae* and encompasses 17 species, whereby only *Y. pseudotuberculosis, Y. enterocolitica*, and *Y. pestis* are known to cause diseases in mammals (Koornhof et al., 1999; Smego et al., 1999). All human pathogenic yersiniae are zoonotic, Gram-negative, facultative anaerobes that are well adapted for survival in a variety of external environments and persistence in various host animals. *Y. pseudotuberculosis* and *Y. enterocolitica* are both enteric pathogens which have emerged within the last 200 million years, whereas *Y. pestis* has evolved as a separate clone from *Y. pseudotuberculosis* about 2000–20,000 years ago (Achtman et al., 1999; Wren, 2003). A recent comprehensive study analyzing over 200 genomes of different *Yersinia* species further demonstrated that human pathogenic *Yersinia* species have evolved by following parallel evolutionary paths: (i) acquisition of similar virulence determinants, e.g., the virulence plasmid pYV that encodes the main virulence genes of *Yersinia* and the chromosomally-encoded cell adhesion and invasion protein Ail, and (ii) functional gene loss and reduced metabolic flexibility (Reuter et al., 2014).

**Figure 1:**
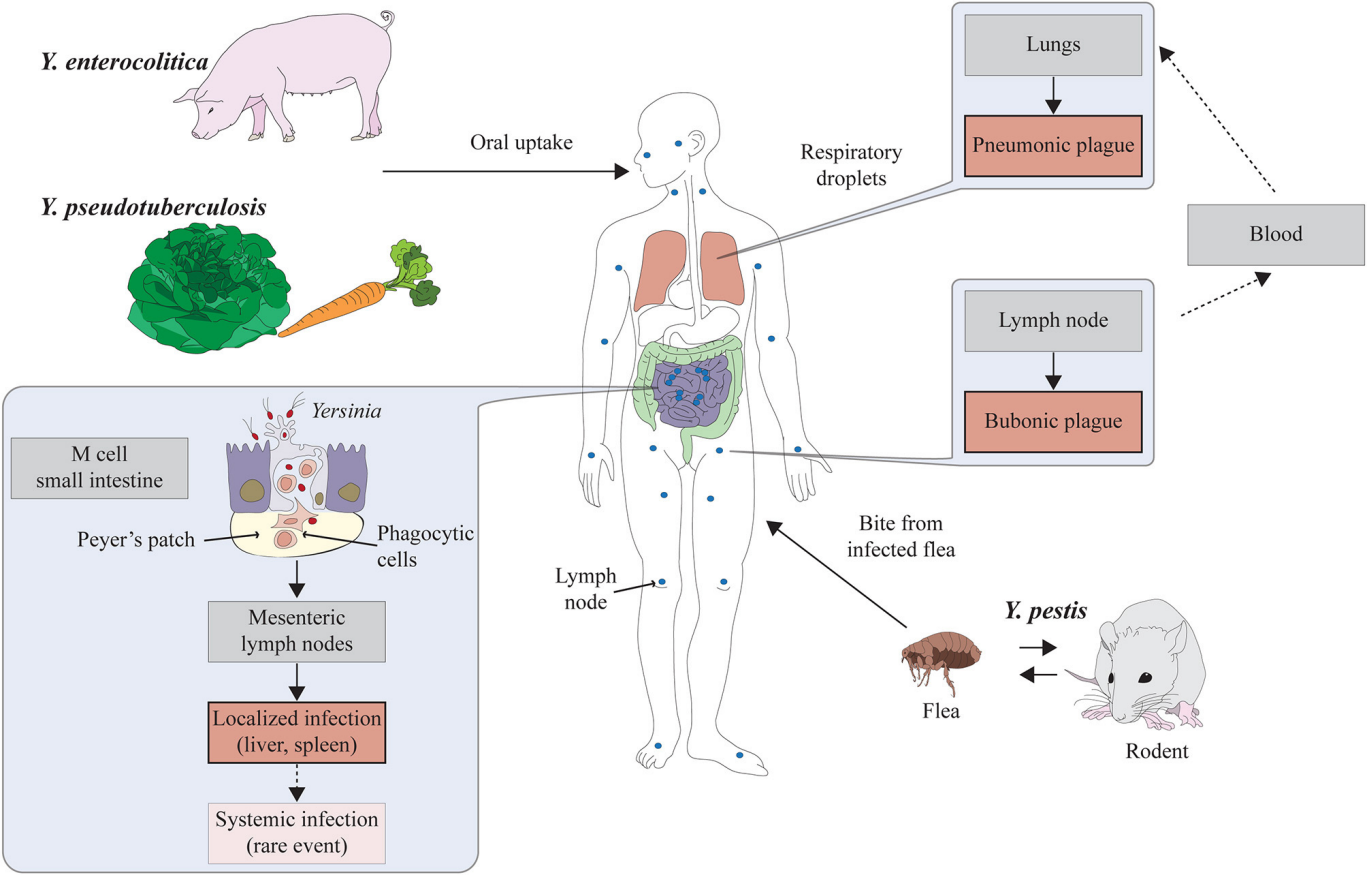
**Lifestyles and pathogenesis of the human pathogenic *Yersinia* species**. The enteropathogenic *Yersinia* species *Y. enterocolitica* and *Y. pseudotuberculosis* are associated with meat (mainly pork) and lettuce/vegetables. They are ingested via contaminated food and enter the lymphatic system through the M cells in the small intestine. The main reservoirs of *Y. pestis* are rodents. Transmission of the bacteria to humans occurs through the bite of an infected flea resulting in bubonic plague. Pneumonic plague is developed when *Y. pestis* reaches the lung and is transmitted via respiratory droplets.

Both enteric *Yersinia* species cause various gut-associated symptoms (e.g., enteritis, ileitis, diarrhea, and mesenteric lymphadenitis) commonly called yersiniosis. Only in very rare cases they can lead to systemic infections and induce extra-intestinal sequelae such as erythema nodosum and reactive arthritis (Koornhof et al., 1999). *Y. pseudotuberculosis* and *Y. enterocolitica* can occupy many different environmental habitats and have been isolated from ground water, soil, plants, and insects. In addition, certain domestic and wild animals were shown to be reservoirs for enteropathogenic *Yersinia* species (Fredriksson-Ahomaa et al., 2006; Fredriksson-Ahomaa, 2012). Both enteric *Yersinia* species are transmitted via the fecal-oral route. Undercooked pork meat is considered to be the major infection source of *Y. enterocolitica* (Bottone, 1997), and vegetables and lettuce for *Y. pseudotuberculosis* (Figure 1).

*Y. pestis* is the causative agent of plague. It is unique in its choice of host habitats and primary mode of transmission. *Y. pestis* generally resides within the lymphatic system, blood, or tissues of rodents and is transmitted to other mammals through direct contact or the bite of an infected flea, when the bacteria are regurgitated from the proventriculus into the dermis during the flea blood meal (Perry and Fetherston, 1997). During this early stages of the infection, *Y. pestis* replicates within macrophages at peripheral host sites (Perry and Fetherston, 1997). From there, they spread into the draining lymph nodes where they replicate and lead to the formation of buboes (hemorrhagic, swollen lymph nodes), which is the characteristic clinical feature of bubonic plague. Subsequently, *Y. pestis* can disseminate into the blood stream leading to a fulminant systemic infection and fatal septicemia. In rare occasions the infection can progress to pneumonia (pneumonic plague) which enables the bacteria to be transmitted from person-to-person via contaminated droplets (Perry and Fetherston, 1997) (Figure 1).

### Host-pathogen interactions

Both enteric *Yersinia* species are armed with a set of pathogenicity factors that enable the pathogens to efficiently colonize the intestinal tract. To survive the acidic environment of the stomach they both induce the expression of urease, an enzyme that counteracts the gastric acidity by neutralizing low pH with ammonia (Young et al., 1996; Hu et al., 2009). Furthermore, they express diverse membrane-anchored surface adhesins and invasins that contribute to interactions with the intestinal epithelium and promote bacterial passage of the intestinal barrier into deeper tissues. During the early stages of the infection, the bacteria attach to and invade into the specialized microfold epithelium (M cells), overlaying the Peyer's patches (PPs) in the most distal part of the small intestine, the ileum (Grutzkau et al., 1990; Isberg and Barnes, 2001). From the PPs, the bacteria disseminate to the mesenteric lymph nodes (MLNs), or other extra-intestinal tissues such as liver and spleen (Cornelis and Wolf-Watz, 1997; Plano and Schesser, 2013) (Figure 1). The outer membrane protein invasin is the most efficient adhesion and internalization factor of enteropathogenic *yersiniae*. Other homologous Inv-type adhesins (InvB/Ifp, InvC), Ail, the homotrimeric autotransporter adhesin YadA, and the PsaA/Myf fimbriae seem to contribute to the dissemination process (Marra and Isberg, 1997; El Tahir and Skurnik, 2001; Grassl et al., 2003). Certain adhesins are also likely to promote colonization of liver and spleen during later stages of the infection, which in case of *Y. pseudotuberculosis* was shown to occur directly without previous passage of the PPs and the MLNs (Handley et al., 2005; Barnes, 2006). *Y. pestis* carries non-functional copies of the adhesin/invasin genes *invA* and *yadA*, and has lost other genes required for intestinal pathogenesis. Instead, it acquired the pMT1 (pFra) plasmid that enables *Y. pe*s*tis* to replicate in and be transmitted by the flea (Achtman et al., 1999). The adhesins Ail and PsaA are also present and appear to contribute to host-pathogen interactions (Lindler et al., 1990; El Tahir and Skurnik, 2001).

### Resistance against immune responses

*Yersinia* pathogenesis is also tightly correlated with its ability to resist or evade host immune responses. Besides their role in host-pathogen interactions, Ail and YadA were shown to provide resistance against complement killing by binding of the regulator factor H and the C4b-binding protein (Bliska and Falkow, 1992; Pierson and Falkow, 1993; Kirjavainen et al., 2008). PsaA was shown to inhibit phagocytosis most likely by binding lipoproteins that prevent recognition by host cells (Payne et al., 1998; Makoveichuk et al., 2003). In addition, all three pathogenic *Yersinia* species possess a type three secretion system (T3SS) to deliver anti-phagocytic Yop effector proteins into host cells, mainly professional phagocytes (Cornelis and Wolf-Watz, 1997; Plano and Schesser, 2013). The T3SS-Yop apparatus is encoded on the 70 Mb virulence plasmid pYV (pCD1 in *Y. pestis*) and is absolutely required for virulence of all three pathogenic *Yersinia* species. This is based on the fact that the translocated Yop effectors are exotoxins that disable the phagocytic machinery by (i) destabilizing the actin cytoskeleton, (ii) suppression of cytokine production and (iii) induction of apoptosis of phagocytic cells (Viboud and Bliska, 2005; Plano and Schesser, 2013). Another crucial anti-phagocytic factor is the F1 capsule protein (Caf1), which is solely produced by *Y. pestis*. Caf1 promotes transmission by flea bite (Du et al., 2002) and is required for pathogenesis during bubonic and pneumonic plague (Sebbane et al., 2009; Weening et al., 2011). Anti-phagocytic capabilities are acquired by *Y. pestis* during early stages of infection, when the bacteria are internalized by macrophages and neutrophils, and enable extracellular survival of the bacteria later on (Lukaszewski et al., 2005). Acquisition of the *Y. pestis* plasmid pMT1 and pPCP1, encoding the *Yersinia* murine toxin (Ymt) that supports survival in the flea (Hinnebusch et al., 2002), and the omptin-like plasminogen activator protease Pla was critical in the evolution of *Y. pestis* (Kukkonen and Korhonen, 2004; Haiko et al., 2009).

## Link between metabolism and virulence of *yersinia*

It is striking that both enteropathogenic *Yersinia* species, which are more distantly related (<60% nucleotide identity), induce similar gastrointestinal disease symptoms, whereas *Y. pestis* and *Y. pseudotuberculosis* which are very similar genetically (>97% nt identity) exhibit markedly different pathogenicities (Chain et al., 2004; Derbise et al., 2010). The underlying molecular mechanism(s) are still unknown, but in addition to the small differences concerning classical virulence factors, also variations in metabolic functions and/or differences in the regulatory mechanisms implicated in metabolic adaptation could contribute to observed differences in pathogenicity and clinical outcome.

### Nutrient sensing and retrieval

The mammalian gastrointestinal tract can be considered an excellent source of nutrients for enteropathogenic *yersiniae*. Nevertheless, nutrient supply can vary considerably in the different gut sections (Rohmer et al., 2011). Simple sugars such as glucose and galactose, resulting from the degradation of disaccharides (lactose, maltose), and starch are readily absorbed in the proximal part of the mammalian small intestine, whereas polysaccharides from plant cell walls (e.g., cellulose, xylan, and pectin) pass into the distal portion of the gastrointestinal tract. Inflammation and hypoxic conditions induced by the immune response can dramatically change the availability of nutrients (Nizet and Johnson, 2009). Furthermore, the host restricts the pathogen's access to essential ions such as magnesium, manganese, zinc, and iron (Brown et al., 2008; Abu Kwaik and Bumann, 2013; Zhang and Rubin, 2013). Within the digestive tract, *Yersinia* also has to compete with the resident microbiota for resources. The intestinal flora comprises about 10^14^ well-adapted bacteria from more than 400 species, which form a special cross-feeding ecosystem, in which anaerobes degrade complex polysaccharides. Other bacteria such as *Escherichia coli* utilize the simple sugars released as breakdown products (Hooper et al., 2002; Chang et al., 2004; Le Bouguenec and Schouler, 2011). In order to successfully colonize the intestinal tract, *Yersinia* must either grow on non-utilized energy/C-sources or process nutrients much more efficiently to outgrow others. A similar situation will be envisioned by *Y. pestis* during replication in the intestinal tract of fleas.

One prerequisite to succeed in the their hosts is the ability of *yersinia*e to sense available C-sources. In particular carbon catabolite repression (CCR) triggered in response of the availability of readily digestible sugars (e.g., glucose) was shown to modulate metabolism and is used to coordinately control the expression of virulence factors via the cAMP receptor protein (Crp) (see below; Görke and Stülke, 2008; Poncet et al., 2009; Heroven et al., 2012b). Moreover, changes in the supply of amino acids are used to adapt metabolism and regulate virulence via the stringent response through (p)ppGpp (Sun et al., 2009; Dalebroux et al., 2010). Usually other environmental cues, such as temperature, oxygen and ion concentration, and pH are used in combination with nutrient sensing to determine the location of *Yersinia* within the host to properly adjust metabolism and pathogenicity (see below).

### Metabolic functions important for *yersinia* to colonize host territories

All pathogenic *Yersinia* species are chemoheterotrophs, consuming organic molecules for energy and carbon. They possess a complex metabolic system with a full complement of often redundant or alternative catabolic and biosynthetic pathways that render them very flexible and robust against changing nutrient concentrations and genetic manipulations. Early studies revealed that all pathogenic *yersiniae* are able to catabolize glucose by the Emden–Meyerhof and Entner–Doudoroff pathway with a complete tricarboxylic acid (TCA) cycle and a functional glyoxylate bypass (Santer and Ajl, 1955; Mortlock, 1962; Brubaker, 1968; Motin et al., 2004). However, in contrast to the enteropathogenic *Yersinia* species, the glyoxylate shunt is constitutive in *Y. pestis*. Moreover, *Y. pestis* is unable to metabolize hexoses via the pentose phosphate pathway due to the absence of glucose 6-phosphate dehydrogenase (Mortlock, 1962). Furthermore, the bacteria lack the methionine salvage pathway and aspartase (AspA) activity. AspA is essential for the complete catabolism of L-aspartate, which undergoes reductive deamination by AspA to yield fumerate that is recycled into the TCA cycle (Dreyfus and Brubaker, 1978). As a consequence L-aspartate accumulates in the bacteria and is excreted, e.g., during expression of the low calcium response (LCR). This causes a loss of metabolic carbon that otherwise would be conserved as oxaloacetate (Brubaker, 2007; Viola et al., 2008). Other differences are that *Y. pestis* is meiotrophic in the biosynthesis of glycine/threonine, L-valine and L-isoleucine, L-phenylalanine, and L-methionine, as well as in the fermentation of melibiose and rhamnose, and in the urease pathway (Burrows and Gillett, 1966; Brubaker and Sulen, 1971; Sebbane et al., 2001; Brubaker, 2006, 2012). *Y. pseudotuberculosis* and *Y. enterocolitica* depend only on the presence of the aspartic family of amino acids (Brubaker, 1991). As a consequence, *Y. pestis* is more dependent on its host to obtain required nutrients. Interestingly, *Y. enterocolitica*, but not *Y. pestis* and *Y. pseudotuberculosis*, is able to metabolize cellobiose, sucrose, and inositol. It further produces cobalamine (vitamin B_12_) under anaerobiosis and can degrade 1,2-propanediol and ethanolamine by cobalamine-dependent enzymes using tetrathionate as terminal electron acceptor (Reuter et al., 2014). Tetrathionate is produced in the inflamed mammalian intestine, e.g., during a *Salmonella enterica* serovar Typhimurium infection (Winter et al., 2010). All these additional metabolic activities could provide a species-specific competitive growth advantage over the largely fermentative intestinal microbiota.

#### Adaptation of yersinia to virulence-relevant conditions

To provide a more comprehensive view, multiple “omic” approaches were performed with pathogenic *yersiniae in vitro* grown under virulence-relevant conditions to identify metabolic pathways and virulence genes that might contribute to pathogenesis. The first of these studies addressed temporal changes in gene expression during a temperature transition from 26 to 37°C mimicking transmission of *Y. pestis* from the flea vector to its mammalian host (Motin et al., 2004). Transcriptional profiling revealed that about 10% of the chromosomal genes were influenced by temperature of which the majority encodes important metabolic functions. The thermal induced global changes caused an inhibition of glycolysis while terminal oxidation of a variety of carbohydrates, amino acids, and fatty acids known to exist in the host was favored. This suggested that plague bacilli might favor fermentative pathways during slow growth within the flea, whereas they prefer oxidative catabolism during rapid proliferation in mammals (Motin et al., 2004). Another analysis addressing differential protein expression in *Y. pestis* following a thermal upshift also demonstrated that several enzymes involved in sugar metabolism (e.g., α-enolase, phosphoglycerate kinase, glyceraldehyde-3-phosphate (G3P) dehydrogenase) are under thermal control (Chromy et al., 2005). Differential expression of these enzymes suggests that different types/concentrations of carbohydrates are metabolized after temperature transition. The metabolic switch in the utilization of specific sugars in different milieus appears to be crucial to trigger virulence.

A transcriptomic analysis of *Y. pestis* was also performed in human plasma in order to identify genes which are required during septicaemic plague in humans (Chauvaux et al., 2007). The most marked plasma-triggered virulence factors are the pYV-encoded T3SS/Yop apparatus, whereas PsaA fimbriae were downregulated. In addition, several genes related to purine/pyrimidine metabolism were upregulated in plasma at 37°C and support a previous observation that purine metabolism is necessary for *Y. pestis* virulence (Munier-Lehmann et al., 2003). An equivalent study analyzing the transcriptome of *Y. pseudotuberculosis* during growth in human plasma showed that this closely related pathogen switches to consumption of glucose, which is readily available in blood/plasma (about 7 mM). Phosphotransferase system (PTS)-encoding genes, glycolysis and phosphoenolpyruvate (PEP)-dependent systems were found to be upregulated (Rosso et al., 2008). This is reminiscent to the “glucose overflow metabolism” channeling the carbon flow toward acetate formation instead of citrate formation to prevent accumulation of NADH. In other *Enterobacteriaceae* such as *E. coli*, acetate accumulation is supported by a simultaneous repression of the glyoxylate shunt, but this is not the case for *Y. pseudotuberculosis*. In the opposite, the *aceBAK* genes encoding the key enzymes of the glyoxylate shunt are upregulated (Rosso et al., 2008), suggesting a need for this species to limit acetate overloads. The constitutive expression of the *aceBAK* operon in *Y. pestis* indicates that derepression of the glyoxylate shunt might also be important for the plague bacilli.

#### Metabolic pathways that contribute to pathogenicity

In the last few years, several studies were published analyzing the *in vivo* transcriptome of *Y. pestis* in the flea vector (Vadyvaloo et al., 2010) and in the mammalian host (Lathem et al., 2005; Sebbane et al., 2006; Liu et al., 2009). Several genes involved in the metabolic adaptation to the different niches as well as classical virulence genes known or predicted to be important for *Yersinia* colonization in the respective host or for resistance against the innate immune response were found to be upregulated.

**Figure 2:**
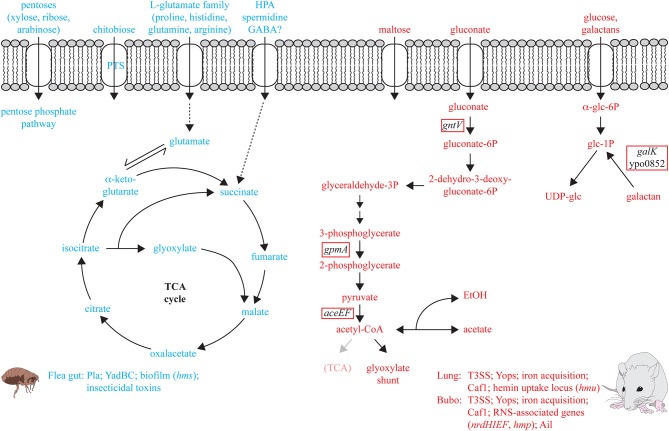
**Metabolic pathways and virulence factors of *Y. pestis* induced in the flea and the mammalian host**. Metabolic functions and pathogenicity traits upregulated *in vivo* are illustrated which are considered to be important for colonization of the flea gut (in blue) and the lung or bubo of the mammalian host (in red). The red box marks genes shown to be required for full virulence of *Y. pestis* in the bubo of infected rats (Pradel et al., 2014).

The *in vivo* transcriptome of *Y. pestis* in the proventriculus of infected fleas revealed numerous metabolic genes involved in the adaptation to the flea gut (Vadyvaloo et al., 2010). Flea meals appear to consist primarily of proteins and lipids with low amounts of carbohydrates. Thus, it is not surprising that mainly amino acids, in particular the L-glutamate group (e.g., glutamine, histidine, arginine, proline) are catabolized by *Y. pestis* in the flea vector (Figure 2). Degradation of these amino acids results in an increased flux of the amino acid carbon through the TCA cycle, the enzymatic genes for which are highly induced in the flea (Vadyvaloo et al., 2010). In contrast, catabolism of hexoses seems not to be important. The glucose PTS is only slightly increased and most other sugar uptake systems are repressed. An exception is the PTS uptake and utilization system for chitobiose, a C-source that is present in the flea's proventriculus spines (Figure 2). During growth in the digestive system of the flea synthesis of most important virulence factors, e.g., the T3SS/Yop apparatus, the iron sequestration systems Ybt, and Yfe, the virulence regulator RovA, and PsaA fimbriae are repressed. However, other crucial pathogenicity genes (e.g., *pla, yadBC*) and insecticidal-like toxin genes are upregulated (Figure 2). Expression of these genes is critical for dissemination from the extravascular tissue at the fleabite site and seems to preadapt *Y. pestis* to resist mammalian innate immunity by acquisition of a phagocytosis-resistant phenotype. This may enhance plague pathogenesis in the very early stages while the full set of thermal controlled virulence factors is still not produced (Vadyvaloo et al., 2010). Also genes of the *Y. pestis hms* operon which are required for the formation of the poly-*N*-acetylglucosamine (PNAG) surface carbohydrate, a major component of biofilms, were found to be induced at moderate temperature and within fleas. Thus, *hms*-dependent biofilms were assumed to support colonization of the proventriculus and facilitate transmission of plague bacteria (Hinnebusch et al., 1996; Vadyvaloo et al., 2010). However, a recent report showed that in two other fully virulent *Y. pestis* strains PNAG synthesis is maximal at 37°C, indicating that this factor may also have a role during mammalian infection (Yoong et al., 2012).

To better understand host-pathogen interactions, adaptation of *Y. pestis* to its mammalian host was further investigated by *in vivo* gene expression profiling in the bubo in a rat model as well as in the lung of a murine pneumonic infection model (Lathem et al., 2005; Sebbane et al., 2006; Liu et al., 2009). Adaptation of the bacterium to the environments encountered within infected mouse lungs includes the induction of genes involved in amino acid biosynthesis (e.g., histidine, pyruvate, glutamate, and aspartate). Of note is also down-regulation of the TCA cycle and the ATP-proton motive force. Simultaneously, iron acquisition systems, the hemin uptake operon, the antiphagocytic F1 protein capsule (Caf1), as well as the T3SS/Yop apparatus are strongly induced, indicating a role in pneumonic plague development (Lathem et al., 2005; Liu et al., 2009) (Figure 2). Iron (Fe), zinc (Zn), and manganese (Mn) ions are structural or catalytic cofactors in many proteins involved in several crucial processes including regulation of gene expression, oxidative stress resistance, and production of virulence genes (Scrutton et al., 1971; Campbell et al., 2007; Ammendola et al., 2008; Ortiz de Orué Lucana et al., 2012). The ability of the host to limit access to these divalent cations has been recognized as a crucial component of the host defense against invading pathogens, and it is well known that high-affinity Fe, but also of Zn and Mn uptake systems are crucial for the establishment of a successful infection by pathogenic yersiniae. In particular acquisition of Fe has been studied for quite some time and the role of metal divalent cations for *Yersinia* pathogenesis was topic of several reviews in recent years (e.g., Carniel, 2001; Rakin et al., 2012; Perry et al., 2012a). In addition to the yersiniabactin (Ybt) biosynthetic or transport genes, several other Fe transporters (e.g., YfeABCD, FeoABC) were shown to contribute to virulence (Heesemann et al., 1993; Bearden et al., 1997; Perry et al., 2012a). Systems known to be involved in Mn uptake also include the Yfe transporter and MntH, and the ZnuABC transporter constitutes a high-affinity Zn uptake system (Desrosiers et al., 2010; Perry et al., 2012a,b; Bobrov et al., 2014). Expression of the transport systems were shown to be upregulated *in vivo* in *Y. pestis* and this supports their role in pathogenesis, but modest effects of (multiple) transporter mutants further suggest that there are additional not yet identified transport systems that also contribute to this process (Sebbane et al., 2006; Desrosiers et al., 2010; Perry et al., 2012a,b; Pradel et al., 2014).

The Caf1 capsule, Ail, and the T3SS/Yop machinery belong also to the most highly expressed known virulence factors of *Y. pestis* in the rat bubo (Sebbane et al., 2006). In contrast to the mouse lung, *Y. pestis* in the bubo is exposed to reactive nitrogen species (RNS) released by polymorphonuclear neutrophils (PMNs). Expression of the *nrdHIEF* operon encoding for the ribonucleotide reductase and *hmp* which encodes a flavohemoglobin that detoxifies RNS was highly increased and required for full virulence of *Y. pestis* (Sebbane et al., 2006; Pradel et al., 2014). In contrast, *Y. pestis* is not or to a lesser extent exposed to reactive oxygen species (ROS) stress in the buboes (Sebbane et al., 2006). In a recent study, a *Y. pestis* mutant library of genes upregulated during bubonic plague in the bubo of rats was constructed and their importance for virulence in a rodent model of bubonic plague investigated (Sebbane et al., 2006; Pradel et al., 2014). About 40% of the mutants that were affected in virulence encoded for metabolic genes. Attenuation most likely reflects reduction of *in vivo* growth due to the loss of a specific metabolic pathway. The results further suggest that *Y. pestis* depends mainly on carbohydrates as C-source (i.e., glucose, galactans, gluconate, and maybe maltose) (Pradel et al., 2014) (Figure 2). The galactans and glucose are most likely channeled toward UDP-glucose synthesis and not to glycolysis, as deletion of the first two upper genes in the glycolysis pathway (*pgi, pfkA*) did not impact *Y. pestis* virulence (Pradel et al., 2014). Virulence mutant testing also supported previous results suggesting that gluconate is an important C-source of *Y. pestis* in its mammalian host (Motin et al., 2004; Pradel et al., 2014). It is likely that gluconate is metabolized to glyceraldehyde-3-phosphate (G3P), pyruvate, acetyl-CoA and acetate, as enzymes of the terminal part of the glycolysis (*gpmA, aceEF*) were essential for competition with the wildtype *in vivo* (Figure 2). Additional observations that (i) deletions of TCA cycle genes such as *gltA* (encoding citrate synthase), *acnA* (acotinase A), and *fumC* (fumarase C) did not affect virulence, that (ii) most of the genes of the TCA were downregulated *in vivo* (Sebbane et al., 2006), and that (iii) the glyoxylate shunt is constitutively expressed, strongly suggest that plague bacilli shift to anaerobic respiration or fermentation during colonization of rodents. Another recent study used transposon mutagenesis and high-throughput sequencing (Tn-seq) to probe the *Y. pestis* genome to detect genes contributing to virulence in mice following intravenous injection (Palace et al., 2014). More than 30 genes with roles in nutrient acquisition and metabolism (e.g., purine biosynthesis, aromatic amino acid biosynthesis) were found to be required for fitness of *Y. pestis in vivo*. Several candidates were also identified by Tn-seq probing of *Y. pseudotuberculosis* (Crimmins et al., 2012). Some identified genes were further shown to be important for *Y. pestis* fitness in the *in vivo* competition experiment by Pradel et al. However, also considerable differences were observed between both *Y. pestis* studies which may be explained by a higher infection dose used by Palace et al.

### Coregulation of metabolism and virulence

Transmission from an environmental/vector-associated lifestyle into the intestine/lymphatic tissues in mammals demands rapid adaptation not only of virulence gene expression but also of metabolic pathways to ensure maximal fitness and competiveness required for pathogen colonization. Sensing of surrounding nutrients/metabolites is an important mechanism signaling the arrival of the pathogen in a certain location within the host and is used to regulate metabolic functions in tight coordination with virulence traits. To endure frequent variations in the nutrient composition *yersiniae* possess a large variety of sophisticated sensing, signal transduction and regulatory strategies to react to abrupt and pronounced changes of the C-source composition. Over the last decades mainly regulatory proteins have been characterized with respect to metabolic control, but lately more and more post-transcriptional control mechanisms implicating small non-coding RNAs have been identified as additional elements controlling virulence and metabolism.

#### Transcriptional control by global regulators

Bacterial two component systems (TCS) are able to sense external stimuli and convert them into a cellular response, typically by controlling expression of multiple metabolic, but also virulence-associated genes. Some TCS have been reported to influence metabolism and virulence in *Yersinia*; among them is the PhoP/PhoQ system.

The pleiotropic TCS PhoP/PhoQ constitutes one of the most crucial signal transduction systems controlling bacterial virulence. It is composed of the membrane-bound sensor kinase PhoQ that responds to low magnesium, low pH environments and host-secreted cationic antimicrobial peptides (CAMPs) and phosphorylates the cytoplasmic response regulator PhoP (Groisman, 2001). Recent studies have shown that PhoP controls the global carbon storage regulator (Csr) system in *Y. pseudotuberculosis* (see below), and Crp in *Y. pesti*s (see below) (Zhang et al., 2013b; Nuss et al., 2014). The *phoP* gene was found to be upregulated in the flea and shown to be required for a normal foregut-blocking infection (Vadyvaloo et al., 2010; Rebeil et al., 2013). It is likely that the system is activated by CAMPs which are secreted into the flea gut during blood meal (Lehane et al., 1997). Furthermore, the *phoP* gene was found to be induced in the lung in an intranasally challenged plague model in mice (Liu et al., 2009). It has been clearly shown that the PhoP/PhoQ system promotes survival and proliferation in macrophages and neutrophils (Miller et al., 1989; Oyston et al., 2000; Grabenstein et al., 2004, 2006; Groisman and Mouslim, 2006). Furthermore, PhoP of *Y. pestis* was shown to repress synthesis of the pH6 antigen (Zhang et al., 2013a). However, the role of the PhoP/PhoQ system for pathogenesis of the different *Yersinia* species is less clear as conflicting results were obtained with different strains and infection models. A strong attenuation was reported for a *phoP* mutant of *Y. pestis* GB and the *Y. pseudotuberculosis* derivative 32777, but only modest defects have been observed with a *phoP* mutant of *Y. pestis* CO92 and the *phoP*-deficient *Y. pseudotuberculosis* strain YPIII (Oyston et al., 2000; Grabenstein et al., 2004; Bozue et al., 2011; Pisano et al., 2014). This strongly indicated that the impact of *phoP* depends on strain-specific differences that remodel regulation and/or composition of the regulon with different outcomes on the virulence phenotype. In fact, in a recent study we could demonstrate strain-specific variations in the PhoP-mediated influence on the Csr system in *Y. pseudotuberculosis* (Nuss et al., 2014).

**Figure 3:**
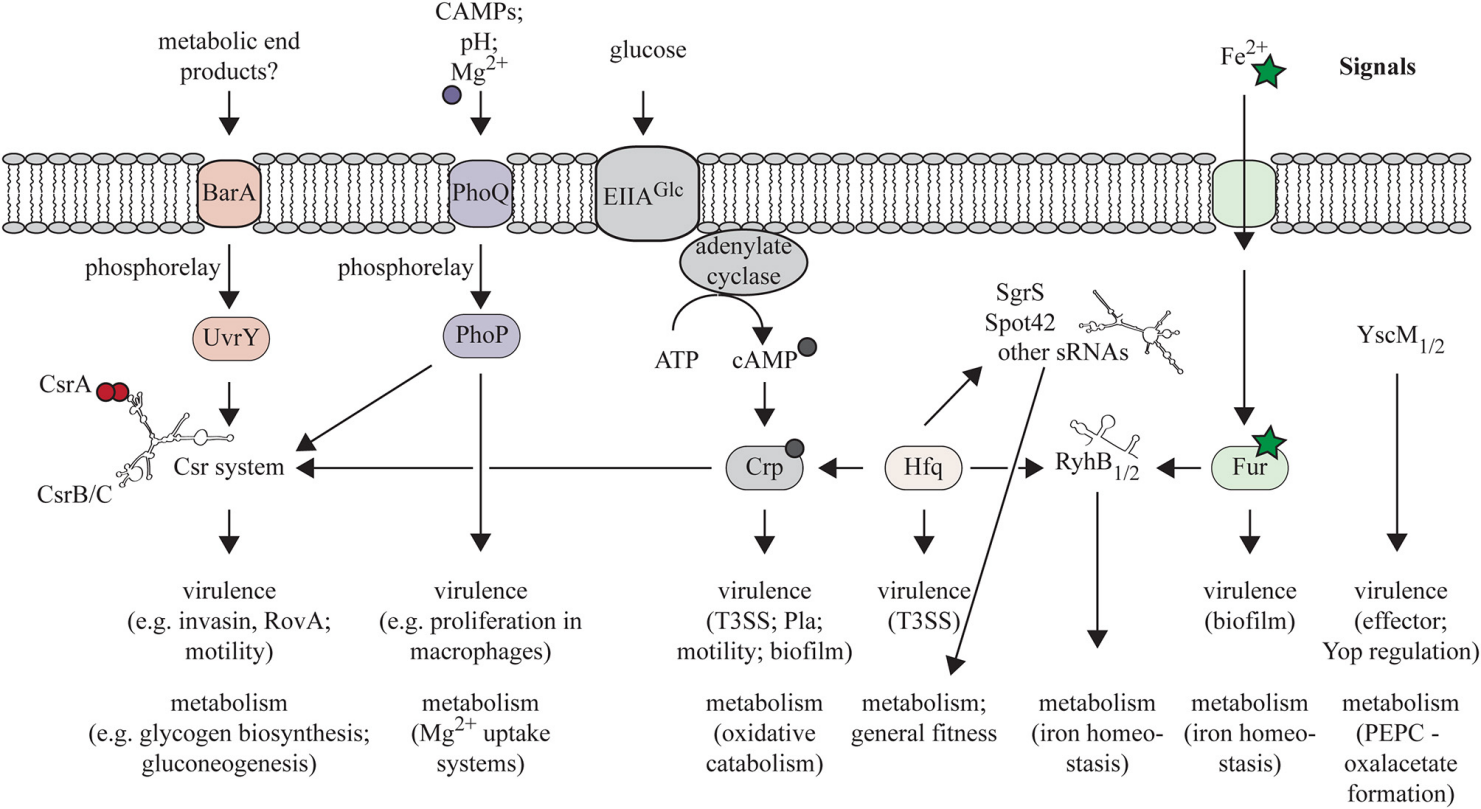
**Schematic overview of regulatory factors that are known to coordinate expression of metabolic functions but also virulence-associated traits in pathogenic *Yersinia* species**. It should be noted that not all regulatory networks have been experimentally verified in all pathogenic *yersiniae*.

Global regulators that govern complex networks and cascades of control elements in a concerted manner achieve coordination of metabolic pathways with pathogenicity mechanisms. One important global transcriptional factor known to control metabolism and pathogenicity in all three human pathogenic *Yersinia* species is Crp. Crp binds the signal metabolite cAMP produced by the adenylate cyclase in the absence of glucose or other efficiently utilizable sugars (Hanamura and Aiba, 1991; Ishizuka et al., 1994). Crp also represses expression of the adenylate cyclase gene *cyaA* (Qu et al., 2013). The cAMP-Crp complex controls at least 6% of the genes in *Y. pestis* and *Y. pseudotuberculosis*, including genes required for growth on different C-sources, survival under carbon, nitrogen, and phosphate limitation as well as virulence (Gosset et al., 2004; Heroven et al., 2012b; Zhan et al., 2008, 2009). In *Y. pestis*, expression of *crp* is crucial for the development of bubonic and pneumonic plague. Most likely this is based on the function of Crp as regulator of the T3SS/Yop machinery and the plasminogen activator protease Pla (Kim et al., 2007; Liu et al., 2009; Zhan et al., 2008, 2009; Lathem et al., 2014). In *Y. enterocolitica*, a *crp* mutant strain was shown to be strongly attenuated in an oral infection model, and Crp-mediated influence on the expression of the flagellar, Ysc/Yop, and the Ysa T3SS is anticipated to contribute to loss of virulence (Petersen and Young, 2002). Similarly, Crp is required for colonization and/or persistence of *Y. pseudotuberculosis* in the MLNs and organs later during infection (Heroven et al., 2012b). In our recent study using comparative metabolomics, transcriptomics and a phenotypic microarray analysis, we could demonstrate that Crp of *Y. pseudotuberculosis* promotes oxidative catabolism of many different C-sources, whereas it represses fermentative patterns. Furthermore, it links carbon metabolism to the regulation of virulence factors via the control of the virulence-associated small RNAs CsrC and CsrB of the Csr system (Heroven et al., 2012b) (Figure 3).

#### Posttranscriptional regulation of adaptation processes

The Csr system constitutes an important global posttranscriptional regulator system. It regulates stability and translation of various mRNAs involved in metabolism and virulence in *Yersinia* and many other pathogens (Timmermans and Van Melderen, 2010; Heroven et al., 2012a). It is composed of the RNA-binding protein CsrA and of two Csr-type sRNAs (CsrB and CsrC in *Y. pseudotuberculosis*) that counteract the activity of CsrA. CsrA acts by binding to conserved (N)GGA motifs in the loop of hairpin structures found close to the ribosomal binding site of the target mRNA thereby affecting translation and/or stability of the transcript. The Csr-RNAs contain multiple CsrA-binding sites and can sequester and inactivate CsrA (Romeo et al., 2012; Heroven et al., 2012a). CsrA has a global influence on the transcriptome of *Yersinia* (Heroven et al., 2012b). The Csr system of *Y. pseudotuberculosis* was first identified to be crucial for the initiation of the infection process. CsrA was found to repress expression of the global virulence regulator RovA that is required for the activation of the primary entry factor invasin and the PsaA fimbriae (Nagel et al., 2001; Cathelyn et al., 2006; Heroven et al., 2008) (Figure 3). In total, about 20% of the CsrA-dependent genes are involved in metabolic processes (Heroven et al., 2012a). The Csr-RNAs seem to be controlled by different regulatory mechanisms in response to ions and availability of C-sources. This is achieved through (i) Crp which positively affects expression of CsrC, and represses CsrB (Heroven et al., 2012b), (ii) the Mg^2+^-dependent TCS PhoP/PhoQ controlling *csrC* transcription (Nuss et al., 2014), and (iii) the TCS BarA/UvrY shown to induce *csrB* (Heroven et al., 2008). The UvrY/BarA system is activated by metabolic end products (format, acetate) in *E. coli* and by an imbalance of the TCA cycle in *Pseudomonas* (Takeuchi et al., 2009; Chavez et al., 2010). The signals to which the *Yersinia* BarA/UvrY system responds are unknown. However, expression of *uvrY* from *Y. pestis* in the lung, but not in the liver and spleen of infected mice, indicate that metabolites/ions present in a certain host niche are able to induce this TCS during infection (Liu et al., 2009).

Another important regulator of post-transcriptional processes in many bacterial species is the small RNA-binding chaperone Hfq. It forms a hexameric ring complex that enables Hfq to simultaneously bind more than one RNA molecule and facilitates binding of sRNAs to their cognate mRNA to strengthen interactions. Binding of Hfq can either stabilize or promote degradation of mRNA transcripts (De Lay et al., 2013). A recent study comparing the global transcriptome and proteome response of *Y. pseudotuberculosis* and *Y. pestis* grown under physiologically relevant temperatures demonstrated that gene and protein expression of conserved virulence factors such as the Yop effector proteins is higher in *Y. pestis* than in *Y. pseudotuberculosis*. In contrast, regulation of metabolism and of the translational machinery seems to underlie a conserved posttranscriptional control. Among these are proteins of the purine and pyrimidine metabolism, glycolysis/gluconeogenesis, pyruvate metabolism, the TCA cycle, and amino-acyl tRNA biosynthesis (Ansong et al., 2013). All these pathways are controlled by the RNA chaperone Hfq in *S*. Typhimurium (Sittka et al., 2007, 2008; Ansong et al., 2009). As Hfq of *Yersinia* has also been shown to play an important role in the regulation of metabolism and general fitness, it is very likely that Hfq controls similar metabolic functions in *Yersinia* (Geng et al., 2009; Bai et al., 2010; Schiano et al., 2010; Kakoschke et al., 2014). Hfq and sRNAs also contribute to virulence of all pathogenic *Yersinia* species, and one important fact is that they are implicated in the posttranscriptional regulation of T3SS/Yop machinery in *Y. pestis* and *Y. pseudotuberculosis* (Geng et al., 2009; Schiano et al., 2010, 2014). Hfq influence seems to occur through Crp. Hfq was recently shown to be required for efficient synthesis of Crp via an unknown positive posttranscriptional mechanism that involves the 5′ untranslated region (UTR) of the *crp* mRNA (Lathem et al., 2014) (Figure 3). Furthermore, the *Y. pestis hfq* mutant is unable to form biofilms in the proventriculus of infected fleas and thus contributes to flea transmission (Rempe et al., 2012).

The implication of sRNAs in the regulation of cellular metabolism has become increasingly recognized (Michaux et al., 2014). Several sRNAs conserved between *Enterobacteriaceae* have been characterized over the last years and their molecular function and targets have been identified mainly by studies performed with *Salmonella* and *E. coli*. Multiple conserved sRNAs were also recently discovered in *Y. pestis* and *Y. pseudotuberculosis* using deep sequencing approaches; among them are SgrS, Spot42, GcvB, and RyhB. The SgrS RNA is involved in a phenomenon called “phosphosugar stress” (Morita et al., 2004). Glucose uptake via the PTS system resulting in accumulation of high levels of generated glucose 6-phosphate is toxic for many *Enterobacteriaceae*. Under these phosphosugar stress conditions, the transcriptional activator SgrR is activated and induces among others the synthesis of the sRNA SgrS. Base pairing of SgrS with the *ptsG* mRNA results in the degradation of *ptsG* and therefore in less uptake of glucose through this glucose-specific permease (Papenfort et al., 2013). Furthermore, the SgrS RNA encodes a small peptide (SgrT), which also helps to rescue *E. coli* from phosphosugar stress by inhibiting glucose transporter activity at the posttranscriptional level. The base-pairing function of SgrS is conserved in *Y. pestis* and *Y. pseudotuberculosis*. However, the sRNA does not produce SgrT as the 5′ end is truncated, indicating differences in the control of phosphosugar stress in *Yersinia* (Wadler and Vanderpool, 2007, 2009; Horler and Vanderpool, 2009). Another conserved sRNA identified in *Y. pestis* and *Y. pseudotuberculosis* is Spot42 (Koo et al., 2011; Beauregard et al., 2013), which is also involved in the regulation of sugar metabolism in *E. coli*. The synthesis of galactokinase (GalK), a protein encoded by the *galETKM* operon important for the conversion of galactose to glucose 1-phosphate is repressed in the presence of glucose. Repression is mediated by binding of the Crp-dependent Spot42 sRNA to the leader of the *galK* mRNA which prevents its translation (Beisel and Storz, 2011). The sRNA GcvB in *S. enterica* serovar Typhimurium and *E. coli*, is primarily expressed under high glycine concentrations and prevents translation of transcripts encoding peptide and amino acid transport systems (Urbanowski et al., 2000; Sharma et al., 2007, 2011; Pulvermacher et al., 2009). In *Y. pestis* KIM6, GcvB possesses two different termination sites leading to two distinct sRNAs. They repress *dppA*, a periplasmic binding component of a major peptide transport system. Deletion of *gcvB* has pleiotropic effects resulting in reduced growth rates and altered colony morphology (McArthur et al., 2006). Furthermore, GcvB was one of the most abundant sRNAs identified in *Y. pestis* under *in vitro* conditions, although the implications of this are still unknown (Koo et al., 2011). In addition, *Yersinia* encodes two RyhB homologs. In *E. coli* and other *Enterobacteriaceae*, RyhB is a key player for adaptation to iron-limiting conditions. It prevents the synthesis of non-essential iron-containing proteins and leads the induction of iron-scavenging siderophores (Massé and Gottesman, 2002; Massé et al., 2007). The RyhB RNAs are highly expressed in *Y. pestis* within infected lungs (but not in the spleen). However, loss of both RyhB variants had no obvious effect on the dissemination capacity and survival of the bacteria after subcutaneous and intranasal infection. It has been assumed that this is due to redundant iron uptake systems (Deng et al., 2012, 2014; Yan et al., 2013).

#### Posttranslational regulation of adaptation processes and virulence regulation

A striking observation of the multi-omic approach by Ansong et al. (2013) was the difference of certain metabolites, in particular glutamate, between *Y. pestis* and *Y. pseudotuberculosis*. As this difference was not reflected at the level of transcription nor on the level of protein synthesis, these results implicate that also post-translational mechanisms are involved in modulating the metabolism *in vivo*. In fact, a recent study investigating the regulation of the T3SS in *Y. enterocolitica* showed that components of the secretion machinery are able to directly interfere with metabolic enzymes. The two homologous proteins YscM1 (homolog of LcrG in *Y. pestis* and *Y. pseudotuberculosis*) and YscM2 regulate expression of the Yop effector proteins. Under non-secretion conditions YscM1/YscM2 repress the synthesis of the Yops. This suppression is relieved when both proteins are secreted upon cell contact (Pettersson et al., 1996; Stainier et al., 1997). Furthermore, they are able to bind and inhibit the function of the *Yersinia* phosphoenolpyruvate carboxylase (PEPC) (Schmid et al., 2009). Under virulence-relevant conditions, PEPC replenishes the oxaloacetate pool in the TCA cycle. Mutants in *yscM1* and *yscM2* displayed increased rates of (i) pyruvate formation via glycolysis or the Entner–Doudoroff pathway, (ii) oxaloacetate formation via the TCA and (iii) amino acid biosynthesis. This indicates that both Yops are involved in the repletion of central carbon metabolism. Modulation of PEPC activity might be important for the metabolic adaptation process of *Yersinia* during the infection. The authors proposed a “load-and-shoot” cycle: In order to prepare the bacteria against the phagocytic attack, PEPC is active and replenishes the TCA cycle, as amino acid synthesis is required (loading). During cell contact, the pre-synthesized Yops are rapidly secreted (shooting). As maintaining the energy charge is more needed than biosynthesis during this process, PEPC is inhibited resulting in a shut down of anaplerosis (Schmid et al., 2009). This cross-talk between T3S and metabolism is further supported by the observation that secretion of the Yop effectors is activated by the amino acids glutamate, glutamine, aspartate and asparagine, feeding into the TCA cycle (Lee et al., 2001).

## Future aspects

The analysis of bacterial metabolism specific to infection is most important to fully understand bacterial pathogenesis to design more effective therapies against pathogens. Use of novel technologies (e.g., deep sequencing, ^13^C isotopologue profiling and phenotype microarrays) has significantly increased our knowledge about the complex crosstalk between the primary metabolism and virulence in bacteria. However, current knowledge of host-adapted metabolic functions is still limited since most results are derived from “omic” data obtained *in vitro* under different virulence-relevant conditions, but not during infection. Thus, future efforts are needed to define growth conditions in the infections sites, tackle utilized C/N sources and identify essential metabolic pathways in the different stages of the infection, e.g., by establishment of *in vivo* “omics” and fluxome analysis. Furthermore, high-throughput screens aimed to identify metabolic genes essential for infection were often performed with a single strain isolate and a particular animal model. Based on observed strain-specific variations and different types, routes, and animals used for the infection obtained results cannot easily be generalized and requests a comparative analysis of multiple strains per species. Other important issues that need to be addressed are: (i) how certain metabolic traits confer a fitness advantage for enteric *Yersinia* when faced with different gut commensals or certain host defense mechanisms, (ii) how do *yersiniae* interfere with the carbon and nitrogen metabolism when they are internalized in host cells, and (iii) how gained information can be exploited to develop novel antimicrobial therapies based on the interference with host-adapted metabolic pathways. Interference with global regulators (e.g., CsrA, Crp, Hfq) or blockage of essential metabolic pathways is an attractive but still unexploited way of controlling plague and other fatale diseases of related pathogens.

### Conflict of interest statement

The authors declare that the research was conducted in the absence of any commercial or financial relationships that could be construed as a potential conflict of interest.
